# Re-Docking Scheme for Generating Near-Native Protein Complexes by Assembling Residue Interaction Fingerprints

**DOI:** 10.1371/journal.pone.0069365

**Published:** 2013-07-16

**Authors:** Nobuyuki Uchikoga, Yuri Matsuzaki, Masahito Ohue, Takatsugu Hirokawa, Yutaka Akiyama

**Affiliations:** 1 Department of Physics, Chuo University, Bunkyo-ku, Tokyo, Japan; 2 Grand Challenge Applications Project for Life Sciences, Next-Generation Integrated Simulation of Living Matter, Computational Science Research Program, Riken, Wako, Saitama, Japan; 3 Department of Computer Science, Graduate School of Information Science and Engineering, Tokyo Institute of Technology, Meguro-ku, Tokyo, Japan; 4 Japan Society for the Promotion of Science, Tokyo, Japan; 5 Education Academy of Computational Life Sciences, Tokyo Institute of Technology, Meguro-ku, Tokyo, Japan; 6 Computational Bioinformatics Research Center, AIST, Koto-ku, Tokyo, Japan; Russian Academy of Sciences, Institute for Biological Instrumentation, Russian Federation

## Abstract

Interaction profile method is a useful method for processing rigid-body docking. After the docking process, the resulting set of docking poses could be classified by calculating similarities among them using these interaction profiles to search for near-native poses. However, there are some cases where the near-native poses are not included in this set of docking poses even when the bound-state structures are used. Therefore, we have developed a method for generating near-native docking poses by introducing a re-docking process. We devised a method for calculating the profile of interaction fingerprints by assembling protein complexes after determining certain core-protein complexes. For our analysis, we used 44 bound-state protein complexes selected from the ZDOCK benchmark dataset ver. 2.0, including some protein pairs none of which generated near-native poses in the docking process. Consequently, after the re-docking process we obtained profiles of interaction fingerprints, some of which yielded near-native poses. The re-docking process involved searching for possible docking poses in a restricted area using the profile of interaction fingerprints. If the profile includes interactions identical to those in the native complex, we obtained near-native docking poses. Accordingly, near-native poses were obtained for all bound-state protein complexes examined here. Application of interaction fingerprints to the re-docking process yielded structures with more native interactions, even when a docking pose, obtained following the initial docking process, contained only a small number of native amino acid interactions. Thus, utilization of the profile of interaction fingerprints in the re-docking process yielded more near-native poses.

## Introduction

Prediction of protein-protein docking is one of the most important approaches for understanding the protein-protein interaction networks of living cells. Among all the approaches, the rigid-body docking method is most useful for the large-scale prediction of protein-protein interaction networks. Since the rigid-body docking process needs input of data from the three-dimensional (3D) structural information of proteins, this approach is suitable to meet the increasing demands for gathering tertiary structural information of proteins [1]. The rigid-body docking process, which is the first step in searching the structure of a native complex, generates many candidate protein complexes, referred to as decoys [2], [3]. A set of these decoys generally includes many structures that are, by far, different from the native structure. Therefore, these decoy sets were further searched to identify the near-native decoys of the protein complex.

The most serious problem encountered in a docking process is that the resulting decoys do not always include the native complex. In the case of rigid-body docking of unbound protein structures, about 55% of the 176 benchmark test cases contained one near-native decoy among 1000 decoys [4]. Even among the bound-state monomer-monomer protein-pairs listed in the ZDOCK benchmark dataset ver.2.0 [5], 3 out of 44 protein pairs did not have any decoys with<5 Å root mean square deviation (RMSD), and one pair did not have any decoys with<10 Å RMSD. Among these protein-pairs was a pair that had undergone large conformational change upon complex formation and was categorized as ‘Difficult’, whereas the other pairs, none of which exhibited large conformational changes, were categorized as ‘Rigid-body’. These results seem to suggest that near-native decoys could not be obtained simply by searching for docking spaces all over the protein surface. To solve this problem, we explored for suitable docking spaces by using selected decoys that were generated from an initial docking process. We reasoned that even though the structure of a decoy is far removed from the native complex structure, it may contain few interactions similar to the native ones. Thus, if enough number of native interactions could be assembled, then it might be feasible to obtain near-native decoys by searching around the areas of assembled interactions. Therefore, in this study, we performed re-docking after assembling interactions of the decoys that were generated from the initial-docking process.

Generally, cluster analysis is used to search for near-native decoys. One of the popular parameters for calculating similarities between the decoys is RMSD, which is useful for comparing 3D-structures. However, RMSD values often depend on the method or algorithm used for the superposition of 3D-structures. We, therefore, developed another profile-based method. Profile- or motif-based methods have already been used in various aspects of bioinformatics. For example, in PSI-BLAST, the query-related sequences are searched by abstracting a position-specific score matrix [6]–[8]. Profile-based methods have been extensively used for examining various types of molecular interactions, such as drug design by virtul screening and protein-ligand docking, mainly involving interactions between proteins and small molecular weight ligands [9]–[21]. Application of these methods to examine protein-protein interactions, though important, is however lacking despite the availability of large amount of data on protein structures. When protein complexes are studied in detail, 3D-coordinates of their composing atoms are used for data analysis. Although a profile-based method is not suitable for observing details of protein-protein interactions, it is useful for analyzing large-scale data of protein complexes. To calculate similarities between the protein complexes, we added interaction fingerprints (IFP) to the post-docking analysis of the protein rigid-body docking process [21]. As a scale for measuring unique similarities between the complex structures, IFP takes into consideration the number of atoms in the interacting amino acid residues of each protein. Accordingly, such a profile-based method could easily evaluate similarities between the molecular complexes, for example, by using Tanimoto coefficient of IFP (TCIFP) [9], [10]. IFP can be applied to molecules with large conformational changes (such as calmodulin), because IFP simply compares between the corresponding residue pairs. Thus, when multiple NMR structures of calmodulin were used as the input structures in the rigid-body docking process, clusters obtained by the IFP method exhibited smaller scattering of energy scores than those obtained by the RMSD method [21].

**Figure 1:**
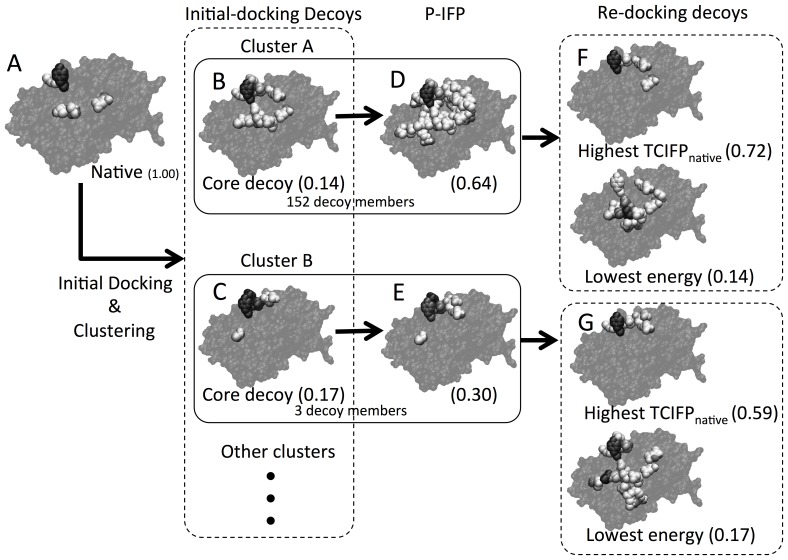
Interaction sites of representative decoys. After the initial-docking process, many decoys were generated from the native 3D structure (A) of cytochrome c peroxidase (PDBID: 2PCC). Core decoys: (B) decoy with lowest energy score and (C) decoy found in the cluster of most number of near-native decoys after the re-docking process. P-IFP was generated from a cluster of decoys as described in the Materials and Methods section (D and E). For every re-docking, only two decoys are shown – one with highest TCIFP_native_ energy and the other with lowest energy (F and G). Frequency of interaction is shown using open and shaded (different shades of black) spheres. A dark black sphere represents the most frequently interacting residue.

The IFP method could be used not only for comparing decoy interactions but also for assembling IFPs corresponding to each interacting residue pair because this profile is additive. After an initial docking process was carried out using the native complex (for example, see Figure 1A), some decoys are found to contain interacting residues similar to the native interactions; such decoys are useful in reducing the search-spaces for docking.

Even though the interactions found in the individual decoys have less similarities to the native interactions (Figures 1B and 1C), it is possible to obtain surfaces that are more similar to the natives than the core decoys after assembling appropriate decoys (Figures 1D and 1E). When some of the assembled IFPs have relatively large fraction of native interactions, we could obtain near-native decoys following the re-docking process (Figures 1F and 1G of “highest TCIFP_native_”). Calculation of TCIFP_native_ is described in detail in Materials and Methods. These processes could be applied to protein pairs for which the initial docking process have failed to generate any near-native decoys. Intrinsically, the docking analysis is for solving complex structures of unknown protein pairs using their unbound-state structures. However, here we have focused on analyzing cases for which near-native decoys were not found in the bound-state structures. To solve this problem, we proposed a method of assembling IFPs of decoys, and applied this method to the bound-state protein 3D-structure datasets.

## Results and Discussion

### Docking Process and Cluster Analysis

After the rigid-body docking process, 29 +/- 28.86 near-native decoys were obtained for 36 out of 44 protein pairs. A near-native decoy is defined as a decoy with interactions similar to the native ones, evaluated using the Tanimoto Coefficient (TCIFP_native_) values of more than 0.4, as detailed in the Material and Methods section. There were 8 protein pairs that did not yield any near-native decoys (Table 1) even in their bound-state forms; these protein pairs were composed of 6 ‘Rigid-body’ type, one ‘Medium-Difficulty’ type, and one ‘Difficult’ type. These categories are detailed in the Materials and Method section. In order to circumvent this problem, we used the profile method for analyzing a set of docking decoys. Accordingly, cluster analysis was performed on all 2000 decoys obtained from the initial protein-protein docking process. After hierarchical clustering of decoys by unweighted pair group method with arithmetic mean (UPGMA), decoys were divided into several groups according to different H-threshold values. The average number of clusters obtained for each protein pair was 537.8 +/- 316.8 in H6, 338.0 +/- 303.8 in H5, and 212.4 +/- 295.9 in H4. Formation of large number of clusters suggested that each cluster was composed of fewer decoys.

**Table 1:** The number of near-native decoys, near-native core decoys, and near-native profile of interaction fingerprints.

PDBid^a^	nnDCY^b^		nnC-DCY^c^	Cluster^d^	nnP-IFP^e^				PDBid	nnDCY		nnC-DCY	Cluster	nnP-IFP			
					T0	T4	T5	T6						T0	T4	T5	T6
1AK4	13	H4	1	85	9	9	9	9	1AVX	7	H4	0	99	10	10	10	10
		H6	4	328	28	28	28	28			H6	2	483	46	46	46	45
1AY7	85	H4	0	48	4	4	4	4	1B6C	24	H4	1	102	10	10	9	11
		H6	5	197	16	16	16	15			H6	6	465	85	85	85	86
1BUH	0	H4	0	111	6	5	4	5	1BVN	1	H4	0	116	6	6	6	6
		H6	0	457	34	34	34	34			H6	1	607	17	17	18	20
1CGI	47	H4	2	125	12	12	10	9	1D6R	4	H4	1	70	7	7	8	8
		H6	16	544	51	51	51	51			H6	1	331	33	33	33	33
1DFJ	0	H4	0	248	1	1	1	3	1E6E	20	H4	3	234	17	13	14	14
		H6	0	750	8	8	8	8			H6	7	703	38	38	38	38
1E96	10	H4	1	65	7	7	5	4	1EAW	81	H4	33	719	79	79	80	80
		H6	4	259	11	11	11	11			H6	41	835	97	97	98	99
1EWY	18	H4	7	1126	28	27	28	28	1F34	0	H4	0	1510	9	9	9	9
		H6	11	1363	39	39	39	40			H6	0	1639	9	9	9	9
1FC2	130	H4	0	49	4	3	3	3	1FQJ	1	H4	1	969	2	2	2	2
		H6	2	207	47	47	46	46			H6	1	1301	2	2	2	2
1GCQ	25	H4	0	15	4	3	2	3	1GHQ	0	H4	0	130	2	2	2	2
		H6	1	84	13	13	13	12			H6	0	524	7	7	7	7
1HE1	6	H4	0	62	8	8	8	8	1KAC	1	H4	0	132	3	3	3	2
		H6	4	236	20	20	20	19			H6	0	550	9	9	9	9
1KTZ	7	H4	0	48	3	2	2	2	1KXP	1	H4	1	343	7	6	5	6
		H6	3	246	13	13	13	13			H6	1	920	16	16	16	16
1KXQ	20	H4	7	114	2	8	8	8	1MAH	4	H4	2	265	5	5	5	6
		H6	12	465	9	15	15	15			H6	2	790	23	23	23	22
1PPE	4	H4	1	76	5	5	6	6	1QA9	34	H4	1	62	5	5	5	5
		H6	2	276	19	19	19	18			H6	10	280	23	23	23	23
1SBB	0	H4	0	165	2	2	1	0	1TMQ	9	H4	0	207	8	9	8	6
		H6	0	622	8	8	8	7			H6	2	722	24	24	22	20
1UDI	2	H4	0	147	3	2	5	5	2BTF	11	H4	2	115	5	6	6	6
		H6	2	590	11	11	11	11			H6	4	409	38	38	38	39
2PCC	0	H4	0	73	3	4	3	4	2SIC	64	H4	3	104	7	6	6	7
		H6	0	281	19	19	19	22			H6	15	471	31	31	31	31
2SNI	1	H4	0	134	6	6	5	5	7CEI	355	H4	3	35	11	11	10	8
		H6	1	525	17	17	17	16			H6	16	136	57	57	55	54
1ACB	47	H4	1	49	10	9	10	6	1GRN	61	H4	0	78	7	6	6	5
		H6	6	226	87	87	87	87			H6	13	371	45	45	45	45
1HE8	0	H4	0	114	1	1	1	1	1I2M	70	H4	4	134	11	9	8	6
		H6	0	353	2	2	2	2			H6	18	576	31	31	31	31
1M10	12	H4	0	141	7	7	8	7	1WQ1	18	H4	1	269	14	14	14	12
		H6	2	501	34	34	34	34			H6	9	855	32	32	32	32
1ATN	53	H4	3	183	6	6	6	4	1FQ1	13	H4	1	119	11	11	10	10
		H6	9	626	19	19	19	19			H6	4	459	39	39	39	39
1H1V	0	H4	0	115	7	7	8	7	1IBR	11	H4	2	269	7	7	7	8
		H6	0	390	35	35	35	36			H6	6	855	20	20	20	20

a PDB ids,

b number of near-native decoys,

c number of near-native core decoys,

d number of clusters, and

e number of near-native P-IFPs

The underlined PDB-ids are the cases where no nnDCYs were found. In this Table, only the results for the H4 and H6 threshold conditions are shown.

As summarized in Table 1, out of 88,000 ( = 2,000 x 44) decoys, we found the following number of near-native decoys in each H-threshold group: 242 in H6 (in 35 protein pairs), 144 in H5 (in 31 protein pairs) and 82 in H4 (in 23 protein pairs). In the higher H-threshold groups, clusters containing larger numbers of clusters containing near-native core decoys were obtained. In the H6 group, all protein pairs with near-native decoys, excluding 1KAC, yielded near-native core decoys. Core decoys were chosen from every group after classifying the decoys into groups according to various H-thresholds. In this work, core decoys were defined as the decoys with lowest energy scores, as re-scored by ZRANK, in their respective groups. Naturally, there were no near-native core decoys when there were no near-native decoys, because core decoys were selected from only 2000 decoys. Among all the H-threshold groups, there were cases where protein pairs with near-native decoys did not have any near-native core decoys because lowest energy scoring near-native decoys were not found. We found 12 such cases in H4 and only one case in H6.

We also performed cluster analysis using root mean square deviation (RMSD) for measuring similarities between the decoy interactions. For our analysis, we used L_RMSD, which is the RMSD between the ligand molecules after the receptor molecules are superimposed using C-alpha atoms. In this case, a near-native decoy was defined as the decoy whose L_RMSD was less than 5.0 Å compared to the interacting component of the native complex structure. We found 200 near-native decoys in 41 protein pairs, and on the average 1375.7 +/- 434.7 clusters were found after classifying the decoys into groups using L_RMSD = 5.0 Å, indicating that the number of decoys in each group was smaller than that obtained using the profile method. For example, classification of decoys into groups using L_RMSD = 10.0 Å resulted in 553.0 +/- 284.6 clusters, a number similar to that obtained using H6 (see above). We found 1187 near-native decoys with L_RMSD value<10.0 Å; this number was comparable to the number of near-native decoys (i.e., 1270) with TCIFP_native_ value ≥ 0.4.

### Obtaining P-IFPs Containing High Fraction of Native Interactions by Assembling Clustered Decoys

**Figure 2:**
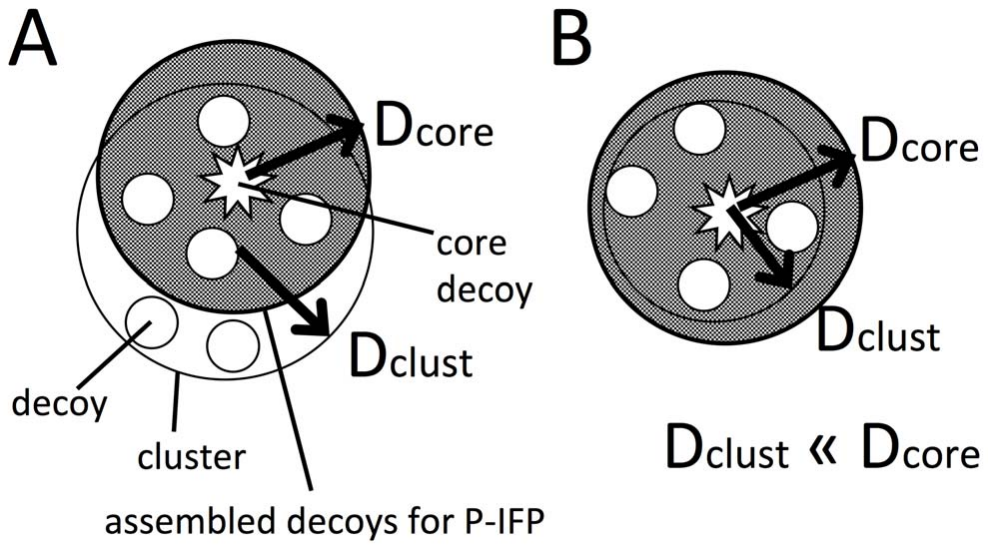
Images of decoys assembled for generating P-IFPs at various H- and T-threshold conditions. Diagramatic representation of decoys (small circles) in a cluster (large circle) at a given threshold condition: unshaded large circle, H-threshold condition; shaded large circle, T-threshold condition. P-IFPs were generated using the decoys in the shaded large circle. D (distance) values used in the distance matrix for cluster analysis are: D = 1 – TCIFP, no similarities; D_clust_ = 1 – T-threshold; and D_core_ = 1 – H-threshold. (A) Some of the decoys assembled in the cluster did not generate any P-IFP. (B) When D_clust_<<D_core_, all decoys assembled in the cluster generated P-IFPs.

After obtaining the core decoy, defined as the decoy with the lowest energy score in a cluster, we generated profile of interaction fingerprints (P-IFPs) by assembling decoys at various T-threshold values, as detailed in the Materials and Methods section. Figure 2 illustrates the concept of T-threshold. After classifying the decoys generated from the initial-docking process, core decoys were selected in terms of energy scores after the decoys were re-scored by using ZRANK. Core decoys (indicated using star marks in Figure 2) were considered as the decoys representing each group. However, one core decoy does not contain enough information for performing the re-docking process. Therefore, to perform re-docking, we needed to assemble information of decoys near a core decoy (decoys in shaded large circle of Figure 2) for each group. The process of assembling decoys for generating P-IFPs are described under “Cluster analysis for selecting core decoys and assembled IFPs” in the Materials and Methods section. Assembled decoys were selected on the basis of near to a core decoy in measure of similarity distance using TCIFP. For selecting assembled decoys, some decoys were not used for generating P-IFPs (Figure 2A). For example, largest numbers of decoys used for generating P-IFPs were assembled using H6 and T4 threshold conditions, as the corresponding D_core_ values were much larger than the D_clust_ values (Figure 2B). On the other hand, the D_core_ values were much smaller than the D_clust_ values when H4 and T6 threshold conditions were used, and thus, only few P-IFP-generating decoys (smallest number) were assembled. Table 2 lists the number of decoys that were assembled for generating P-IFPs at various thresholds. The number of assembled decoys increased as the H-thresholds increased from H4 to H6. Under all H- and T-threshold conditions, each protein pair in the test group generated near-native P-IFPs, defined as TCIFP_native_ ≥ 0.4.

**Table 2:** Number of decoys assembled for generating P-IFPs.

		H-threshold		
		H4	H5	H6
T-threshold	T4	1520.0 +/− 265.7	1848.3 +/− 260.2	1953.8 +/− 227.7
	T5	1097.5 +/− 254.2	1581.1 +/− 249.0	1892.5 +/− 223.1
	T6	671.9 +/− 266.2	1117.5 +/− 240.2	1669.0 +/− 213.4

Average number of decoys and standard deviations were obtained from all clusters under the indicated H- and T-threshold conditions. The T0 values are not shown because in each group all decoys were used for generating P-IFPs.

**Figure 3:**
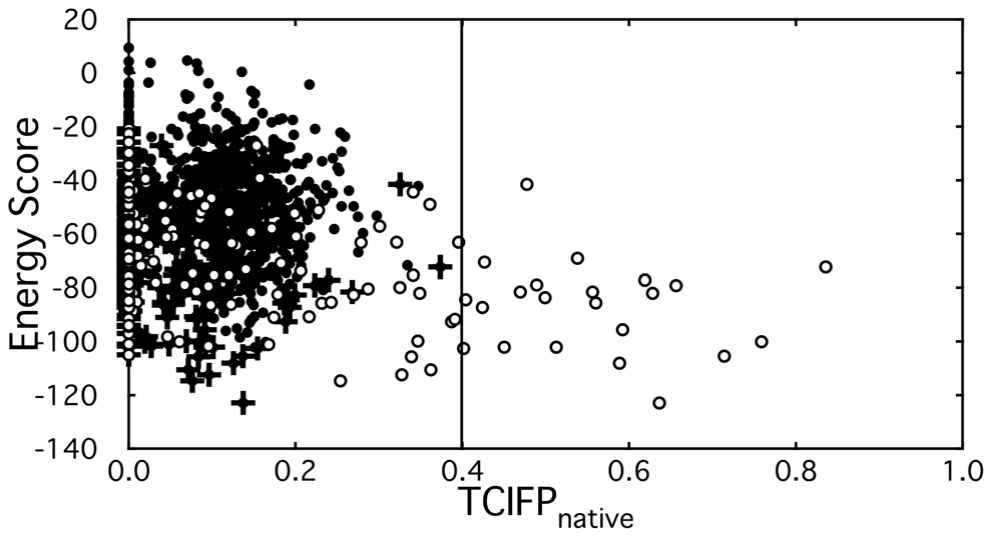
Improvement of TCIFP_native_. Example shown here is for the protein complex between cytochrome c and cytochrome c peroxidase (PDB-id 2PCC). Near-native P-IFPs were obtained for this complex as described in Materials and Methods. This plot shows no near-native decoys in the H6–T6 threshold group. Energy score of P-IFP is defined as the lowest energy of the decoy. Closed circles: 2000 generated decoys, crosses: core decoys, and open circles: P-IFPs.

Results summarized in Table 1 show that all bound-state protein pairs yielded near-native P-IFPs even in cases where there were no near-native decoys generated from the initial docking process. It is notable that near-native P-IFPs were also found even when there were no near-native core decoys. In other words, this procedure yielded more near-native P-IFPs than near-native decoys in 31 protein pairs, suggesting that the P-IFPs might include more native interactions than the individual decoys. For example, Figure 3 shows the changes occurred in the TCIFP_native_s during three steps: generation of decoys from the initial docking process, selection of core decoys and generation of P-IFPs from the assembled decoys. Data plotted in Figure 3 illustrate the case of a protein pair having no near-native decoys and least number of divided clusters. In the first step (i.e., docking process for obtaining 2000 decoys) no data point was found in the area where TCIFP_native_ ≥ 0.4. In the second step, we found that all the core decoys were distributed in the area where TCIFP_native_<0.4, which is natural because the core decoys were chosen from the first set of decoys. In the last step, after generating P-IFPs using properly assembled decoys with a certain T-threshold, we found data points in areas where TCIFP_native_ ≥ 0.4. Some of the P-IFPs, which had TCIFP_native_ = 0.0, were derived from decoys with TCIFP_native_ = 0.0. These results suggest that more native interactions can be obtained by assembling decoys after appropriately choosing the core decoys.

In all 44 protein pairs, 96.1% of near-native P-IFPs had more than 40% native interactions. We then observed the interaction sites in one of the protein complexes shown as an example in Figure 1, and compared the interaction sites of the decoys in the native complex (Figure 1A), core decoys (Figures 1B and 1C) and P-IFPs (Figures 1D and 1E) of the receptor molecule. We found that the frequent interacting residues were same in both core decoys and P-IFPs. Other ‘false-positive’ interaction sites, which are not found in the native complex, were however found in the core decoys and also in P-IFPs (Figure 1B – 1E). Occurrence of such frequent ‘false-positive’ sites resulted in lowering the TCIFP_native_ values of the core decoys (TCIFP_native_ values 0.14 and 0.17). On the other hand, their TCIFP_native_s of P-IFPs were found to be 0.64 and 0.30, which were higher than the TCIFP_native_s of the core decoys (Figure 1D and 1E). In all protein pairs, more than 90% of P-IFPs had higher TCIFP_native_ values than those of the core decoys. Out of 9,345 clusters of H4, percentages of P-IFPs with higher TCIFP_native_ values than the core decoys were as follows: 93.3% in T6, 93.7% in T5, 94.1% in T4 and 94.3% in T0. The highest percentages of P-IFPs with higher TCIFP_native_ values were found among the 23,661 clusters in H6, which were 94.6% in T6 and 94.8% in the other T-threshold cases. Similarly, out of 14,871 clusters in H5, 93.8% to 94.5% of P-IFPs showed higher TCIFP_native_ values than the core decoys. Among all H-threshold cases, the highest percentages (94.3% in H4, 94.5% in H5, and 94.8% in H6) of P-IFPs having higher TCIFP_native_ values were found in the T0 group. These results suggest that it is possible to use P-IFPs with higher fractions of native interactions in the re-docking process for obtaining more near-native protein complex 3D structures.

### Analysis of IFP Similarity with Natives

**Figure 4:**
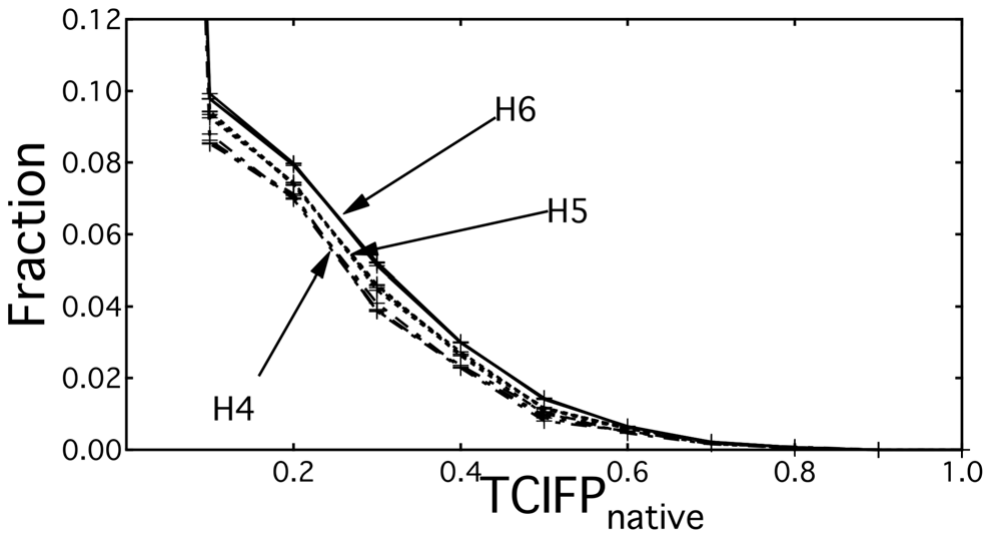
Distribution patterns of TCIFP_native_s of P-IFPs at different H- and T-threshold conditions. TCIFP_native_s of P-IFPs for all 44 protein pairs were calculated using the indicated H- and T-threshold conditions. At a given H—threshold condition, all T-threshold conditions produced the same line plot.

Because interaction sites of P-IFPs are used for the re-docking process, they are related to the docking search spaces. The number of interaction bits in a P-IFP depends on a set of assembled decoys. After dividing the decoys into clusters, if a set of decoys with much varied interaction sites is used for generating a P-IFP, the number of interaction bits tends to be large. The number of bits is related to the TCIFP_native_ values, and it depends on the balance between the native complex and P-IFP. When the numbers of bits in two profiles are largely different, TCIFP range is restricted [22]. Next, we analyzed the distributions of TCIFP_native_ of P-IFPs to determine how the number of interaction bits in P-IFP varies with respect to H-threshold. As shown in Figure 4, the distribution patterns of TCIFP_native_s of P-IFPs at various T-thresholds were very similar under the same H-threshold condition (see also Table 1), which suggested that the T-thresholds have little influence on obtaining high TCIFP_native_ values. We found higher fraction of TCIFP_native_ in the H6 group than in the H5 and H4 groups. Since P-IFPs with higher TCIFP_native_s were obtained under higher H-threshold condition, only the higher H-thresholds generated better size of interaction bits in P-IFPs. Accordingly, when the H-threshold value was set to H7 or H8, most clusters were composed of single decoys, indicating that at these high H-threshold conditions most P-IFPs were same as the IFPs of core decoys. Thus, P-IFPs generated under high H-threshold conditions (such as H7 and H8) are not expected to generate enough docking search spaces. This result suggests that a suitable H-threshold condition, which is H6 in this study, could indeed be obtained. However, this was not the case with the T-thresholds, because in Figure 4 we obtained almost identical plots at all T-thresholds conditions for each H-threshold. Thus, we could not determine any reasonable T-threshold condition from these results. Therefore, we decided to use H6 and T6 conditions in the re-docking process.

### Re-docking Process Generated more Near-native Decoys than the Initial Docking Process

**Figure 5:**
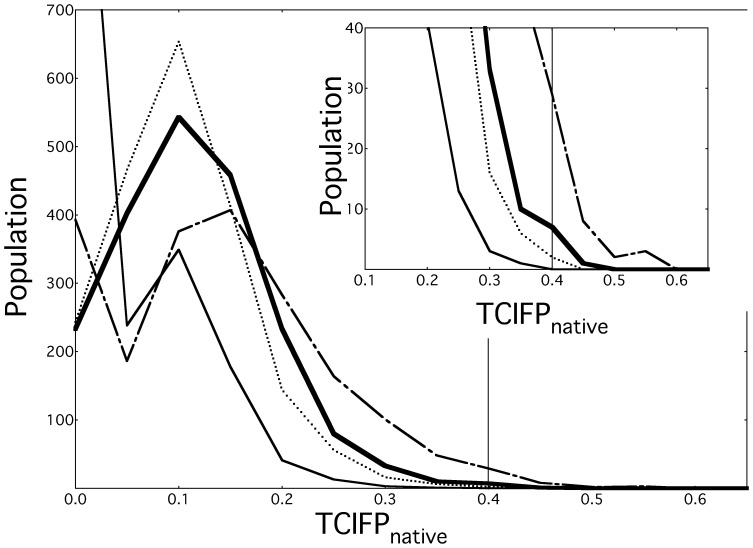
Distributions of TCIFP_native_s of decoys. A bound-state protein pair, PDB-id 2PCC, was used for this analysis. TCIFP_native_ values were determined as described in the Materials and Methods. Thin solid line, 2000 decoys generated by the initial-docking process; dotted line, 2000 decoys generated by the re-docking process using the near-native P-IFP with highest TCIFP_native_ (0.84); thick solid line, 2000 decoys generated by the re-docking process using the near-native P-IFP with lowest energy score; dash-dot line, 2000 decoys generated by the re-docking process using the P-IFP with most (41.7%) native interactions. We found 42 near-native decoys in the last case.

We obtained near-native P-IFPs for all bound-state protein pairs, even in cases where there were no near-native decoys. This result, however, was not enough to solve the rigid-body docking problem since we still could not obtain the 3D-structures of protein complexes because of the abstract nature of P-IFP. Nonetheless, near-native P-IFP provides a very informative profile for selecting the docking space area. This is illustrated in Figure 5, which shows the distributions of TCIFP_native_s of decoys. In the bound-state of the protein pair that was used for analysis in this example (i.e., PDB-id 2PCC), there were no near-native decoys, but there were near-native P-IFPs (see Table 1 and Figure 5). We performed three re-docking processes for comparison: first one used near-native P-IFPs with low energy scores, second one used near-native P-IFPs with highest TCIFP_native_s, and third one used P-IFPs with most native interactions. In these cases, we obtained more decoys with higher TCIFP_native_ than those from the initial-docking process, even though each one of these re-docking processes yielded different distribution patterns (Figure 5). Thus, we obtained largest number (i.e., 42) 3D-structures of near-native decoys when near-native P-IFPs with most native interactions were used in the re-docking process. In Figure 1, we showed actual interaction sites for two core decoys. After the re-docking process, we obtained decoys with higher TCIFP_native_s than those of the corresponding core decoys and P-IFPs (Figures 1F and 1G). We also found that the resulting decoys with lowest energy scores were almost same TCIFP_native_s as the corresponding core decoys.

Next, to determine which P-IFPs generated more near-native decoys by re-docking, we randomly selected 12 protein pairs (1ACB, 1AK4, 1ATN, 1AY7, 1B6C, 1BVN, 1D6R, 1GCQ, 1GHQ, 1GRN, 2PCC, and 7CEI) and subjected them to the re-docking process. Results summarized in Table 3 (columns 2–4) show that we were able to obtain near-native decoys after the re-docking process even when no near-native decoys were found by the initial-docking process. In the initial-docking process, only 2000 decoys were generated. However, as the re-docking process uses multiple input data (P-IFPs) generated from several groups of decoys, we could explore the docking space more efficiently by the re-docking process than by the intial docking process. Therefore, it is possible to solve the sampling problem of rigid-body docking by performing re-docking using P-IFPs. Even when no near-native decoys were found in the initial-docking process (1GHQ and 2PCC), which could be considered as a case of most difficult situation, we were able to obtain near-native decoys by using the re-docking method outlined here. Therefore, the re-docking method is a powerful tool in conditioning non-near-native decoys derived from the decoy sampling that resulted from a rigid-body docking. These results suggested that better decoy sampling was achieved by the re-docking process than the initial docking process, simply because the P-IFPs used in the re-docking process restricted the docking surfaces of protein molecules. Accordingly, when P-IFPs with more native interactions are used in the re-docking process, we expect to obtain more near-native decoys. We next identified the most number of near-native decoy-generating P-IFPs by assessing three P-IFP properties in terms of native interactions: TCIFPnative and two types of ratio of native interaction bits used for the re-docking process – one involving native interaction surface (“nat/nat” in Table 3) and the other involing P-IFP surface (“nat/P-IFP” in Table 3, same as “F_nat_”). For each property, we then ranked the P-IFPs with most number of near-native decoys and the results are summarized in Table 3. In 9 out of 12 cases (1ACB, 1AK4, 1AY7, 1B6C, 1GCQ, 1GHQ, 1GRN, 2PCC, and 7CEI) assessed in terms of F_nat_, we found that the P-IFPs ranked in the top 10 (“nat/P-IFP” column in Table 3, top 10 are underlined), indicating that the P-IFPs with most number of near-native decoys are profiles involving more native interactions (i.e., high F_nat_). It is natural that the P-IFP with more native interactions could generate more near-native decoys. However, F_nat_ values were among the highest for some of them, suggesting that the generation of more near-native decoys not only depended on the high value of F_nat_, but also depended on which of the interacting components of P-IFP (e.g., ‘key residues’ involved in protein interaction) were included in the analysis [23], [24]. In the case of 2PCC, for example, the highest F_nat_ value was actually same as the second highest F_nat_ value. Thus, when selecting appropriate P-IFPs, one could use information on the ‘key residues’.

**Table 3:** Comparison of fractions of near-native decoys and rankings of 12 P-IFPs generating most number of near-native decoys in the re-docking process.

PDBid	fraction of #nnDCYs^a^			#cluster	Ranking of max. #nnDCYs (for each property)		
	init-docking^b^	re-docking			nat/P-IFP ( = F_nat_)	TCIFP_native_	nat/nat^e^
		average^c^	maximum^d^				
1ACB	0.0235	0.0450	0.1655	225	1	85	143
1AK4	0.0065	0.0115	0.2135	327	4	8	97
1ATN	0.0265	0.0178	0.539	625	92	102	108
1AY7	0.0425	0.0467	0.475	196	6	5	11
1B6C	0.012	0.0368	0.1545	464	9	190	175
1BVN	0.0005	0.0150	0.213	606	11	53	37
1D6R	0.002	0.0074	0.0915	330	179	175	9
1GCQ	0.0125	0.0463	0.215	83	7	1	4
1GHQ	0	0.0041	0.3125	523	1	6	3
1GRN	0.0305	0.0553	0.264	370	1	77	178
2PCC	0	0.0008	0.021	280	2	36	24
7CEI	0.1775	0.1584	0.33	135	2	58	72

Fractions of near-native decoys (nnDCYs) are shown in columns 2–4. After calculating each property of P-IFP, we arranged the property rankings in the descending order (columns 6–8). Top ranks in columns 6–8 represent the highest value for the indicated property, suggesting that the corresponding property is useful in obtaining most number of near-native decoys. Underlined ranks are top 10 P-IFPs.

a number of near-native decoys (#nnDCYs)

b fraction of #nnDCYs in 2000 decoys generated by the initial-docking process

c fraction of #nnDCYs in all decoys ( = (#cluster)×2000)) generated by the re-docking process

d fraction of #nnDCYs in 2000 decoys of cluster with the most number of nnDCYs.

e fraction of native interactions found in a P-IFP

There is another approach for obtaining near-native decoys in which residues involved in the native interactions are first searched and then the docking is carried out using the interacting residues as the interface. Generally, such ‘key residues’ do not include all the neighbouring residues that are present in the native interacting surface. Therefore, in this approach, it is necessary to specify that the docking search surface include the neighbouring residues. Our method using IFPs, however, can easily assemble protein surfaces necessary for obtaining near-native decoys.

## Conclusion

We proposed a method to generate P-IFP for obtaining near-native interactions by assembling decoys after choosing the core decoys from the decoy clusters that were created using various H-thresholds. Using this method we obtained P-IFPs with high TCIFP_native_ values for all the protein pairs in the dataset of bound-state protein complexes even though some of the protein pairs yielded no near-native decoys. We also proposed a re-docking process in which the P-IFPs were used for confining the docking search space by utilizing the results of the initial docking process. This process could generate 3D-structures of decoys with higher TCIFP_native_ values in the bound-state cases. The number of near-native decoys depended on the interacting components that are shared between the P-IFP and the native interface, suggesting that not all residues included in the native interface are crucial. Therefore, in order to get the near-native decoys, the P-IFPs should possess these crucial interacting components of the native interface. Thus, more studies are needed for generating better P-IFPs to refine this re-docking process for obtaining decoys with higher TCIFP_native_ values.

## Materials and Methods

In this work, re-docking process was performed using the following 4 steps: 1) initial-docking by ZDOCK; 2) generating profiles of IFPs (P-IFPs) after cluster analysis of decoy sets; 3) restricting receptor surface using P-IFP information; and 4) re-docking by ZDOCK. In the first section, dataset and docking options are described. The second section mainly describes the methods for evaluating similarities between the decoys using cluster analysis and for defining near-native decoys. In the third section, definition of core decoys and method for generating P-IFPs are described. The last section contained a description of the re-docking process using P-IFPs.

### Docking Process & Dataset

We selected 44 protein complexes from the commonly used protein-protein docking benchmark 2.0 dataset [25], [26] and used the available data (Table 4) in our study. As each selected protein complex is composed of two monomers, each complex is, therefore, suitable for use in the typical docking process. These 44 protein complexes were categorized as follows: 34 rigid, 6 medium-difficult and 4 difficult protein complexes, each complex consisted of a pair of monomer proteins. These categories are based on the structural differences between the bound and unbound states of these protein complexes [25], [26]. The benchmark dataset was constructed by dividing the protein complex coordinates into single constituent protein coordinates. In this case, data for one complex structure was divided into data from two monomer structures (namely, receptor and ligand). Definitions of receptor and ligand were followed as in the ZDOCK benchmark dataset. 3D-Structural data of the receptor and ligand pairs were fed into the ZDOCK ver.2.3.1 program as the input data [27]. ZDOCK was used with the option for high rotational sampling density of 6 degree (option “-D”). We used 2,000 decoys in this work. We used the same ZDOCK options for the re-docking process. Details of the re-docking process are described under “Re-docking process” (see below).

**Table 4:** List of PDB-ids of 44 protein complex structures selected for this study.

Rigid-body (34)									
1AK4	1AVX	1AY7	1B6C	1BUH	1BVN	1CGI	1D6R	1DFJ	1E6E
1E96	1EAW	1EWY	1F34	1FC2	1FQJ	1GCQ	1GHQ	1HE1	1KAC
1KTZ	1KXP	1KXQ	1MAH	1PPE	1QA9	1SBB	1TMQ	1UDI	2BTF
2PCC	2SIC	2SNI	7CEI						
**Medium Difficulty (6)**									
1ACB	1GRN	1HE8	1I2M	1M10	1WQ1				
**Difficult (4)**									
1ATN	1FQ1	1H1V	1IBR						

### Definition of IFP and Similarity between Decoys

As suggested previously, it is sufficient to compare the interacting fragments rather than the whole structures to obtain information on near-native molecular interactions [9], [10]. Accordingly, profiles of interacting amino acid pairs were obtained using the dimplot command of the LIGPLOT program [28]. For this purpose, we used a LIGPLOT default cut-off distance of 3.9 Å between the non-hydrogen atoms [28]. After the dimplot analysis, IFP was introduced for profiling protein-protein interactions [21]. We used IFPs for comparing decoys. Information on residue pairs was entered into a bit sequence, in which one bit corresponded to a residue pair. If a pair was found, the bit was assigned a numerical value based on the number of interacting atoms; in the case where there was no interacting pair, the bit was assigned a numerical value of zero. At first, we tested a basic concept of similarity between IFPs consisting of only 0 and 1 bit values. After generating an interaction profile of the molecular complex, cluster analysis was performed. Similarity between the decoys and native molecular complexes was determined by calculating the Tversky similarity [29] as follows:
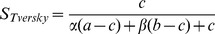
where *a* and *b* are the number of bits including queries *P_a_* and *P_b_*, which are sequences consisting of *a* and *b* numbers of non-zero bits, respectively, and *c* is the common bit-number between *a* and *b*. Parameters α and β varied independently from 1 to 0. When α = 1 and β = 1, the similarity between the queries *P_a_* and *P_b_* could be calculated as follows:



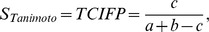
where *S_Tanimoto_* is known as the Tanimoto coefficient (TCIFP). We used TCIFP when comparing decoys to native interactions. IFPs were subsequently used in cluster analysis to compare decoy interactions, which are independent of the method used for the superposition of the 3D-structural data. The TCIFP index was used to quickly calculate whole pairs of decoys. For example in the bit sequence{s}, TCIFP between “0100”( = sequnce A) and “0110”( = sequence B) {is} was calculated {as} to be 0.5 using a = 1, b = 2, and c = 1. When sequence C {is} was “0001”, TCIFP { = } was calculated to be 0 using a = 1, b = 1, and c = 0. When identical sequences are compared, TCIFP = 1.0 using a = b = c. In this study, each element in IFP describes the number of atoms involved in the interaction between a pair of amino acid residues. The following equation was used for calculating TCIFPs:



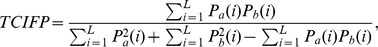



We used the TCIFP_native_ value as the similarity index of the native interaction profile. We paid special attention in calculating the TCIFPs of the receptor proteins, making sure that only the number of atoms in each interacting residue of the receptor protein was used for describing an IFP. Near-native decoys were defined as decoys with TCIFP_native_ values more than 0.4. In this study, decoys were first divided into clusters, and then the corresponding elements were added for generating the representative IFPs for each cluster. Similarity between the IFPs was calculated after normalizing all elements whose values ranged from 0.0 to 1.0. When calculating TCIFP using P-IFPs after assembling the decoys, values of elements in the P-IFPs were normalized.

We also defined the fraction of native interactions in a decoy (F_nat_) as F_nat_ = c/b, which is used in Critical Assessment of Predicted Interactions (CAPRI). In this work, bit number c indicates the number of native interactions in a P-IFP with bit number b. When a P-IFP is identical to the native interaction surface, F_nat_ = 1. This measure is used for calculating the similarity of P-IFPs after assembling the IFPs of decoys, because the P-IFPs were generated by accumulating interactions found in the corresponding bits of a set of decoys. For the re-docking process, it is important to know how many bits of native interactions are in a P-IFP.

### Cluster Analysis for Selecting Core Decoys and Assembled IFPs (P-IFPs)

Cluster analysis of the post-docking data was carried out to search for core decoys. Similarities among IFPs (TCIFPs) of decoys ranged from 0.0 to 1.0, which corresponded to completely different and virtually same, respectively. This TCIFP was then converted into the *D* value, used in the distance matrix of cluster analysis, by using the relationship *D = 1-TCIFP*. We used unweighted pair group method with arithmetic mean (UPGMA) algorithm for the cluster analysis, which is categorized according to a hierarchical algorithm and one of the pair group methods, and is often used for generating phylogenetic tree of life. We used the statistical computing R software ver.2.8.0 for the cluster analysis. To compare results, we divided the decoys using three threshold values of TCIFP: namely, 0.4, 0.5, and 0.6. These parameters, called H-thresholds, were respectively termed as H4, H5, and H6. For example, the parameter H4 implies that each cluster in this group is composed of decoys with TCIFP similarity index of more than 0.4. Similarly, decoys with higher similarities were assembled into clusters in H5 and H6 groups.

To obtain the core decoys in each cluster, we used energy scores, which was calculated as a linear weighted sum of electrostatic, desolvation, and van der Waals energies by using the ZRANK program [30]. In this study, the decoy with the lowest energy score among a group of decoys, after re-scoring using ZRANK, was defined as a core decoy, which seems to be the most stable decoy. Therefore, for each protein pair, the number of core decoys was same as the number of divided groups. If any protein pair did not have any near-native decoys, we could not obtain near-native core decoys for that pair because the core decoys were selected from only 2000 decoys that were generated by the initial-docking process. We generated the interaction profile by assembling decoys that are expected to have native interactions.

Assembled decoys were chosen as decoys similar to a core decoy in a threshold, called a T-threshold (illustrated in Figure 2). The T-threshold conditions T0, T4, T5 and T6 corresponded to TCIFP values 0.0, 0.4, 0.5, and 0.6, respectively. Decoys with higher similarity to the core decoy were assembled for generating IFPs at higher T-threshold conditions. When T0 condition was used, all decoys in a cluster were used for creating the assembly of decoys for generating IFPs, because TCIFP = 0.0 means that the decoys are completely different. As Table 2, not all decoys were used for assembling to generate a P-IFP, excepting for the case of T0. Multiple P-IFPs were generated from one protein pair because cluster analysis using a value of H-threshold produced multiple clusters (groups). For example, in the case of 2PCC, we obtained 280 clusters after cluster analysis using the initial-docking decoys, which in turn generaing 280 P-IFPs. For each cluster, the selected IFPs of decoys were assembled by adding up each and every bit of the corresponding interaction residue pairs. Thus, each bit of a P-IFP indicates an interaction residue pair of the assembled decoy. We expected that when P-IFPs contained more native interactions, more near-native decoys were obtained through the re-docking process.

### Re-docking Process

We performed the re-docking process using P-IFPs. To restrict the surface of the receptor molecule, we used the block function, “block.pl” script, included in the ZDOCK package, which blocked the molecular surface before the start of the docking process [28]. In the re-docking scheme, residues of interacting bit in P-IFPs were used for the as the docking surface, indicating that interaction of other residues were not considered in the re-docking process. Consequently, we obtained decoy sets, which interacted with the restricted surface in terms of bits of P-IFP. We used ZDOCK for obtaining the re-docking decoy sets with the option for high rotational sampling density of 6 degree (option “-D”) and generated 2000 decoys for each protein pair with restricted surface. Because of multiple P-IFPs, when the full re-docking process was carried out for a protein pair, we obtained more than 2000 decoys; for example, in the case of 2PCC, we obtained 560,000 ( = 280 x 2000) decoys.
